# Understanding Lung Deposition of Alpha-1 Antitrypsin in Acute Experimental Mouse Lung Injury Model Using Fluorescence Microscopy

**DOI:** 10.1155/2016/5768312

**Published:** 2016-12-06

**Authors:** Mengmeng Wang, Yutian Zhan, Jianqing Chen, Haojing Rong, Shawn P. O'Neil, Brahma Ghosh, Vuong Nguyen, Jane Owens, Xianfeng Li, Denise M. O'Hara

**Affiliations:** ^1^Pharmacokinetics, Dynamics and Metabolism, Pfizer Inc., Andover, MA, USA; ^2^Drug Safety R&D, Pfizer Inc., Andover, MA, USA; ^3^Clinical R&D, Pfizer Inc., Cambridge, MA, USA; ^4^Molecular Imaging Laboratory, Pfizer Inc., Andover, MA, USA; ^5^Rare Disease RU, Pfizer Inc., Cambridge, MA, USA

## Abstract

Human plasma-derived *α*1-antitrypsin (AAT) delivered by intravenous infusion is used as augmentation therapy in patients with emphysema who have a genetic mutation resulting in deficiency of AAT. Inhalation is an alternative route of administration that can potentially increase the efficacy and convenience of treatment. This study was conducted to determine whether delivery to the lungs, initially via the intratracheal (IT) route of administration, would deliver efficacious levels of a recombinant AAT (rAAT) to the site of action in the lungs in mice. ^125^I-radiolabeled rAAT, fluorophore-conjugated rAAT (rAAT-Alexa488), and NE680 (neutrophil elastase 680, a silent fluorescent substrate of neutrophil elastase which fluoresces in the near-infrared range upon activation by neutrophil elastase) were used to characterize the pharmacokinetics and tissue distribution profile, distribution of rAAT within the lung, and efficacy of rAAT to inhibit neutrophil elastase at the site of action, respectively. The study has demonstrated that rAAT was able to gain access to locations where neutrophil elastase was localized. The histochemical quantification of rAAT activity relative to dose at the site of action provided here will improve confidence in predicting the human dose via the inhalation route.

## 1. Introduction

Human *α*1-antitrypsin (AAT) is a serine proteinase inhibitor produced primarily by hepatocytes, macrophages, and bronchial epithelial cells [1]. The wild type protein is a major elastase inhibitor within the lung [2], where its primary physiological role is to inhibit neutrophil elastase (NE) [3]. More than 120 AAT protein variants have been identified and plasma concentrations of AAT strongly depend on its genotype [4, 5]. One in two thousand North Europeans is homozygous for the Z (342Flu→Lys) variant of AAT, which tends to polymerize and accumulate in hepatocytes [6]. The accompanying AAT plasma deficiency leaves the lungs exposed to neutrophil elastase, resulting in premature emphysema [7], which can be relieved by IV infusion of human plasma-derived AAT [8]. Currently, a number of preparations of purified plasma AAT for IV infusion are on the market (Prolastin (Talecris Biotherapeutics, Research Triangle Park, NC, USA), Aralast (Baxter, Deerfield, IL, USA), etc.); however, high production costs and the inconvenience of IV treatment to the patient are significant drawbacks to this therapeutic approach. It is estimated that only about 2% of AAT administered via the IV route reaches the target organ, the lungs [9]. Administration of AAT by inhalation rather than by the IV route is a potential alternative means of therapy that may reduce the dose required for efficacy while improving the convenience of treatment, because it would capitalize on the enormous surface area of the lung as a potential absorptive surface through which AAT could gain access to the pulmonary interstitium. Inhalation as a route of administration is based on previous findings that droplets of 3 *µ*m in diameter inhaled as an aerosol have the potential to reach the alveolar surface [10, 11]. Preliminary predictive modeling suggested that the site of action is in the pulmonary alveolar interstitium and epithelium lining fluid (ELF) and that delivery of sufficient concentrations of functional AAT to inhibit neutrophil elastase will lead to the rescue of AAT deficiency in the lungs (data not shown). Hubbard et al. [12] have examined the pulmonary absorption of an aerosol of yeast-produced human recombinant *α*1-antitrypsin in sheep and demonstrated that aerosolized AAT was deposited on the epithelial surface and diffused across the alveolar epithelium. However, the study was not able to assess the exact localization of AAT in the lung and whether AAT was able to reach the sites of action in the interstitium and ELF.

A recent imaging study by Kossodo et al. utilized a neutrophil elastase-specific near-infrared fluorescence imaging agent, neutrophil elastase 680 FAST (NE680), which remains optically silent in the nonactivated state but fluoresces upon cleavage by NE, to quantify NE activity associated with lung inflammation [13]. In the study, significantly higher NE680 fluorescent signal was observed in mice with lipopolysaccharide/fmet-leu-phe (LPS/fMLP) induced acute lung injury (ALI) than in healthy controls because intranasally administered LPS and fMLP act synergistically to cause lung inflammation via massive neutrophil infiltration and degranulation [14–16], leading to increased NE activity.

To assess the effective target coverage that leads to efficacy after inhalation of AAT, we conducted a pharmacokinetics (PK) and tissue distribution study in mice using ^125^I labeled recombinant AAT ([^125^I]rAAT). To evaluate pulmonary rAAT concentrations in the LPS/fMLP-induced ALI mouse model, fluorophore-labeled rAAT was administered via the IT route and the magnitude and biodistribution of rAAT were investigated using fluorescence microscopy on lung cryosections. In addition, the functional activity of rAAT delivered to the lung was evaluated by measuring the magnitude of NE680 fluorescence by fluorescence microscopy.

## 2. Experimental Procedures 

### 2.1. Materials

N-Succinimidyl-3-(tri-n-butylstannyl)benzoate (SIB, C23H35NO4Sn; MW = 508.23) was synthesized by Texas Biochemicals Inc. PD-10 column was obtained from GE Healthcare. LPS stock was purchased from InvivoGen (cat.# TLRL-PELPS). fMLP stock was purchased from Sigma (cat.# 47729-10MG-F) and NE680 was purchased from PerkinElmer (cat.# NEV11169). Recombinant AAT was supplied by Pfizer Inc.

### 2.2. Iodination of rAAT and Dosing Solution Preparation

The iodination (^125^I) of rAAT was prepared via SIB coupling chemistry [17]. After labeling, the product, for example, [^125^I]rAAT, was purified by PD-10 column. The protein concentration in the purified radiolabeled rAAT solution was determined by radiometric size exclusion chromatogram (SEC).

The dosing solution was prepared by mixing the unlabeled rAAT with ^125^I-rAAT, in phosphate buffered saline (PBS), yielding a final concentration of 0.5 mg/mL or 3.75 mg/mL for IV or IT groups, respectively. The final dosing solution was prepared 1 day prior to the in-life study and was stored under refrigeration (2~8°C). The dosing solution was brought to ambient temperature prior to administration to the animals.

### 2.3. In-Life PK and Tissue Distribution Studies and Sample Collection

Male Balb/c mice (~8 weeks of age) were purchased from Charles River (Wilmington, MA) and Pfizer Institutional Animal Care and Use Committee approved all aspects of these studies. All studies were performed in accordance with the National Institutes of Health guide for the care and use of animal resources. Mice in each dose group (*N* = 3) received a single dose of [^125^I]rAAT (2 mg/kg via intravenous (IV) route or 5 mg/kg via IT route, based on the most recent body weight measurement) in a volume of 4 mL/kg (IV) or 1.33 mL/kg (IT). At various time points, blood was collected via retroorbital or cardiac puncture into serum separator tubes. The serum was harvested by centrifuging the blood sample at 10000 ×g for 5 min, and protein-associated radioactivity was measured following TCA (trichloroacetic acid) precipitation. For TCA precipitation, an aliquot of 50 *µ*L serum sample was mixed with 150 *µ*L of mouse serum and 200 *µ*L of 20% TCA. After thoroughly mixing, the mixture was kept at ambient temperature for approximately 5 minutes and then centrifuged at 10000 ×g for 5 min. Total radioactivity (cpm) in the sample and the supernatant (as “free label”) were measured in a gamma counter. The physical decay was corrected, and the concentration (ng eq./mL) of [^125^I]rAAT molecules in serum was reported based on the TCA precipitated radioactivity (protein-associated).

At various time points, the tissues of interest were collected after blood sampling and whole body perfusion. The whole body perfusion was conducted with 20 mL heparin/saline solution (147 mg/L heparin sodium salt in saline) per mouse. The tissues of interest (including lung, liver, and kidney) were collected and weighed, and the radioactivity in each tissue specimen was measured in a gamma counter; the physical decay was corrected. The concentration (ng eq./gram) of [^125^I]rAAT in tissue was reported based on its measured total radioactivity (cpm).

### 2.4. Pharmacokinetic Analysis

Plasma pharmacokinetic parameters for [^125^I]rAAT were calculated using noncompartmental methods with the aid of Watson (Version 7.4). Data in the terminal log-linear phase were analyzed by linear regression to estimate the terminal rate constant (*k*) and half-life (*t*_1/2_ = 0.693/*k*). At least the last three time points were used to calculate* k*. Total AUC_inf_ was determined as the sum of AUC_0–last_ and AUC_extra_, where AUC_0–last_ was calculated from 0 to the last time point (*t*_last_) with the last measurable concentration (*C*_last_) using the linear trapezoidal rule and AUC_extra_ is the extrapolated portion of the area from *t*_last_ to infinite using *C*_last_/*k*. Total body clearance (CL) based on plasma concentrations was calculated as dose/AUC_inf_, and the volume of distribution at steady state (*V*_dss_) was calculated as CL*∗*AUMC/AUC, where AUMC is the area under the first moment curve.

### 2.5. Fluorescence Labeling and Characterization of Labeled rAAT

rAAT in PBS-CMF formulation (0.5 mL, 2 mg/mL) was adjusted to pH 9.0 using aqueous NaHCO_3_ (0.5 *μ*L, 1 M). The resultant was transferred to a vial containing Alexa488-carboxylic acid, TFP ester, bis(triethylammonium) salt (Life Technologies) and the reaction mixture magnetically stirred at ambient temperature for 1 hr. At the completion of the reaction, the labeled rAAT-Alexa488 conjugate was purified by spin column chromatography using azide-free Pierce Zeba™ desalting columns (Thermo Fisher Scientific; MW cutoff 7 KDa) with PBS. The conjugate was stored at 4°C, protected from light.

Purity of rAAT-Alexa488 was characterized using SE-HPLC (Column: Agilent SEC-5, 150 Å, 7.8 × 300 mm; Mobile phase: PBS, flow rate: 1.0 mL/min) with detection wavelengths of 280 nm (for protein) and 494 nm (for Alexa488). The dye-to-protein ratio was determined spectrophotometrically (LAMBDA Bio, PerkinElmer).

Functional activity of rAAT-Alexa488 was assessed using rAAT-Alexa488 (30 *μ*L, 1.92 mg/mL), rAAT (9.93 mg/mL), human neutrophil elastase (hNE) from Calbiochem (cat.# 324681), hNE substrate, MeOSuc-Ala-Ala-Pro-Val-AMC from Bachem (cat.# I-1271), 50 mM Tris, 1 M NaCl, and 0.05% (w/v) Brij-35 (pH 7.5) in 96-well black flat-bottomed plates from BD Falcon (cat.# 353241). rAAT-Alexa488 and rAAT were incubated at various concentrations (final concentration up to 100 nM), respectively, with hNE (final concentration 5 nM) at room temperature for 30 min followed by addition of the hNE substrate (final concentration: 1 *µ*M). The mixture was incubated at room temperature for further 15 minutes before fluorescence intensity was read using Ex380/Em460 (EnVision Multilabel Plate Reader, PerkinElmer).

### 2.6. Preparation of LPS, fMLP, and NE680

Five milligrams of rAAT was dissolved in 1 mL ultrapure H_2_O and then further diluted in PBS-CMF to 2.5 mg/mL for dosing. The bulk fMLP stock was made up in ethanol (4.6 mL) and DMSO (10 *µ*L). The dosing solution was prepared by diluting the fMLP stock solution in PBS-CMF to a final concentration of 800 nM. The entire NE680 stock was diluted in PBS-CMF to a concentration of 200 *µ*M. fMLP and NE680 were mixed at equal volumes with final concentrations of 400 nM and 100 *µ*M, respectively.

### 2.7. Animal Study Using Acute Lung Injury Mouse Model

**Figure 1:**
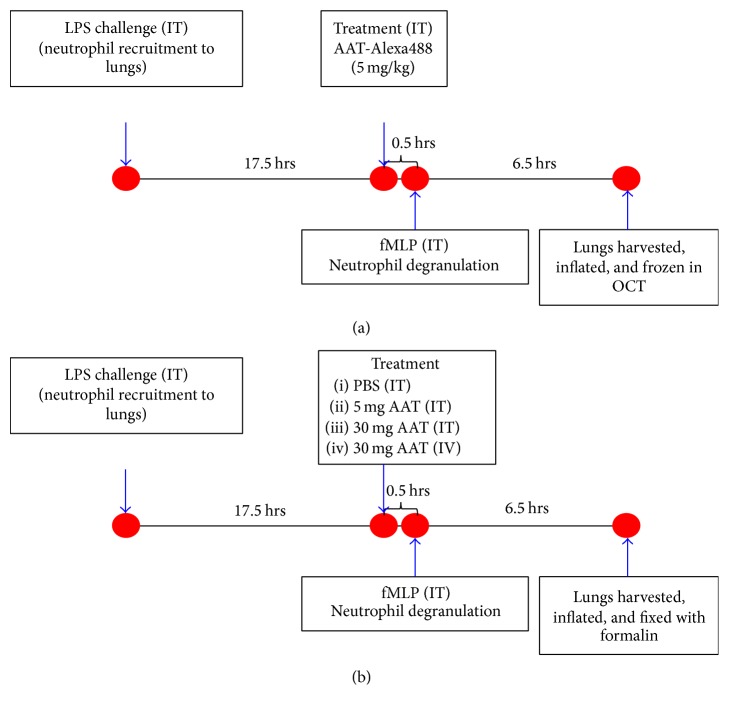
Diagram of the process of (a) Study 1 with rAAT-Alexa488 for biodistribution endpoints and (b) Study 2 with the NE680 probe for analysis of rAAT activity.

Acute lung inflammation (ALI) was induced in mice according to published protocols with slight modifications [13]. Female C57BL/6J mice were purchased from Charles River Laboratories (Wilmington, MA) and used at the age of 8–10 weeks. Process diagrams of the studies are illustrated in Figure 1(a) (rAAT-Alexa488 study) and Figure 1(b) (NE680 probe substrate study). For both studies, mice were challenged on day 1 with 100 *µ*g of LPS in 40 *µ*L PBS via IT route.

#### 2.7.1. ALI Model with rAAT-Alexa488 (Study 1)

As illustrated in Figure 1(a), seventeen and one-half hours after LPS challenge, mice (*N* = 5) received an IT administration of 40 *µ*L of PBS alone or 5 mg/kg rAAT-Alexa488. Eighteen hours after LPS dose, mice received an IT administration of 40 *µ*L of 400 nmol of fMLP.

#### 2.7.2. ALI with NE680 Probe (Study 2)

As illustrated in Figure 1(b), seventeen and one-half hours after LPS dose, animals (*N* = 5) received an IT dose of PBS alone, 5 mg/kg or 30 mg/kg rAAT, or an IV dose of 30 mg/kg rAAT. Eighteen hours after LPS dose, all the animals received an IT dose of 400 nM/4 nmoles of NE680 FAST/fMLP in 40 *µ*L. The naïve group did not receive any administrations.

### 2.8. Sample Collection and Processing

Six and one-half hours after PBS or rAAT-Alexa488 or NE680 FAST/fMLP administration, the animals were anesthetized with isoflurane and exsanguinated prior to tissue harvest.

#### 2.8.1. ALI Model with rAAT-Alexa488 (Study 1)

Harvested lungs were inflated with OCT (Optimum Cutting Temperature) embedding medium and then sectioned into two portions and frozen in a slurry of isopentane and dry ice.

#### 2.8.2. ALI Model with NE680 Probe (Study 2)

Harvested lungs from each animal were inflated with ~10 mL of 10% neutral buffered formalin and the trachea was ligated with suture material to ensure that formalin was retained within the lungs to permit adequate fixation. Formalin inflated lungs were immersed in a container of 10% NBF and fixed for 24 hours prior to wet trimming. The entire lung pluck was processed into a paraffin block.

All lungs were embedded (both paraffin and OCT/frozen) in an orientation such that the long airway of the left lung lobe served as an anatomical landmark for consistency between animals. Three to five 4 *µ*m thick sections were collected from each tissue block for histochemical assays (immunohistochemistry and fluorescence microscopy).

### 2.9. Immunohistochemistry and Fluorescence Microscopy

#### 2.9.1. rAAT-Alexa488 Detection

rAAT-Alexa488 was localized in lung cryostat sections by direct fluorescence microscopy. The specific cellular disposition of rAAT-Alexa488 in mouse lungs was accomplished using a dual fluorescence assay in which ciliated respiratory epithelial cells lining large airways were localized by indirect immunofluorescence for acetyl-*α*-tubulin. Lung cryostat sections were incubated with 1% BSA and 3% normal goat serum in PBS containing 0.1% Triton ×100 at room temperature for 1 hour to block nonspecific antibody binding, followed by incubation with rabbit anti-human acetyl-*α*-tubulin (Lys40) monoclonal antibody (D20G3, Cell Signaling Technology, Danvers, MA) diluted 1 : 100 in PBS containing 1% BSA for 2 hours at room temperature. After washing (3 × 5 minutes) with PBS, tissue sections were incubated with goat anti-rabbit-Alexa594 (Life Technologies) secondary antibody, diluted 1 : 500 in PBS containing 1% BSA for 1 hour at room temperature. Nuclei were counterstained with hematoxylin, and sections were cover-slipped in VECTASHIELD mounting medium with DAPI (4′,6-diamidino-2-phenylindole) (Vector Labs, Burlingame, CA). Immunofluorescent images were acquired using an UltraVIEW confocal microscope (PerkinElmer, Waltham, MA), and* z*-stack images were collected and constructed into 3D images with Volocity software (PerkinElmer) for visualization of rAAT-Alexa488 localization in the lungs.

#### 2.9.2. Neutrophil Immunohistochemistry (IHC)

Neutrophils were identified in paraffin sections of lung tissue from the NE680 probe study using a rat anti-mouse neutrophil monoclonal antibody (clone 7/4, ab53457; Abcam, Cambridge, MA). IHC assays were performed as immunoperoxidase reactions on the Ventana Discovery XT automated immunostainer platform (Ventana Medical Systems, Inc., Tucson, AZ), using Ventana OmniMap detection reagents and the brown substrate/chromogen diaminobenzidine (DAB). All steps in the IHC assays, including deparaffinization, antigen retrieval, blocking of nonspecific reactivity, incubation with primary and secondary antibodies and detection reagents, and hematoxylin counterstaining, were performed on the immunostainer according to Ventana protocols. Whole slide images of lung IHC sections were acquired with a Nanozoomer 2.0 HT (Hamamatsu, Japan), and immunoreactivity of the whole lung section was measured by quantitative immunohistochemistry (QIHC) using Definiens Tissue Studio image analysis software (Definiens, München, Germany). QIHC data were expressed as IHC percent (antibody 7/4-immunoreactive area as a percent of total lung cross-sectional area).

#### 2.9.3. NE680 FAST/fMLP Detection

The tissue distribution of NE680/fMLP was evaluated directly from 5 *µ*m paraffin sections, using a Zeiss Axio Imager fluorescence microscope (Carl Zeiss, Inc., Pleasanton, CA) equipped with a Cy5 filter. Whole slide images were captured with a 10x objective to include acquisition of tissue autofluorescence to facilitate visualization of pulmonary histology. Total NE60 FAST/fMLP fluorescence from an entire lung section from each mouse was quantified using AxioVision software and expressed as percent NE680/fMLP positive area.

#### 2.9.4. Statistical Analyses

Group median values for antibody 7/4 immunoreactivity and percent NE680/fMLP positive area were compared among dose groups using a nonparametric one-way analysis of variance (ANOVA) (Kruskal-Wallis test) combined with Dunn's multiple comparison test. Significant differences were assumed for probability values < 0.05 (*p* < 0.05). All statistical analyses were performed using GraphPad Prism 6.03 software.

## 3. Results

### 3.1. PK and Tissue Distribution of [^125^I]rAAT in Mouse Serum following IV or IT Administration

**Figure 2:**
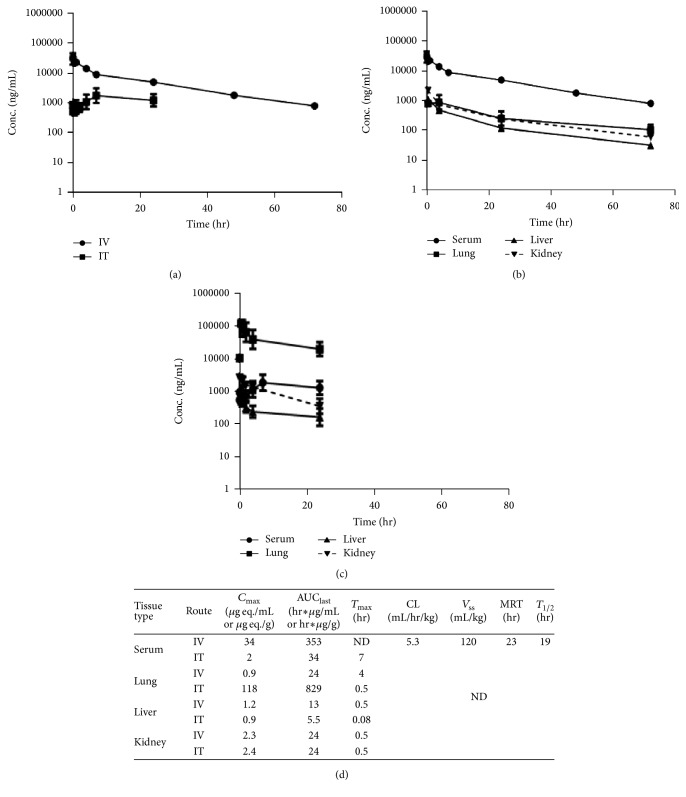
Pharmacokinetics of rAAT. The concentration time profiles of rAAT in serum (a) and tissues (b, c) in male Balb/c mice following a single dose of 2 mg/kg [^125^I]rAAT via IV route (b) or 5 mg/kg via IT route (c). The PK parameters following 2 mg/kg IV administration of [^125^I]rAAT are listed in (d).

The radioactive equivalent concentration (ng eq./mL) of [^125^I]rAAT derivatives in serum was reported based on the TCA precipitated radioactivity. The serum concentrations of [^125^I]rAAT over a 72-hour period after IV or IT dosing of 2 mg/kg are illustrated in Figure 2(a) and the serum PK parameters of [^125^I]rAAT after IV administration are listed in Figure 2(d). After IV administration of 2 mg/kg, the AUC and total body clearance (CL), calculated based on serum concentrations, were 353 hr*∗µ*g eq./mL and 5.3 mL/hr/kg, respectively, and the half-life (*T*_1/2_) was 18.6 hrs. Following 2 mg/kg of IT administration, AUC_last_ was 34 *µ*g*∗*hr/mL and the bioavailability was 10%.* T*_1/2_ via IT route was not determined due to an incomplete PK profile.

Radioactive equivalent tissue concentrations (ng eq./gram) and PK parameters of lung, liver, and kidney after IV or IT administrations of [^125^I]rAAT are listed in Figures 2(b), 2(c), and 2(d). The tissue concentrations decrease at the same rate as serum concentrations, indicating that no accumulation of [^125^I]rAAT occurs in these organs. [^125^I]rAAT exposure in the lung was significantly higher following IT versus IV administration, while drug exposures in liver and kidney are similar after IT or IV dosing.

### 3.2. Characterization of rAAT-Alexa488

The rAAT-Alexa488 conjugate was characterized using SE-HPLC and spectrophotometric measurements. No unconjugated rAAT or unbound Alexa488 were observed by SEC (data not shown). The Alexa488 : rAAT ratio was 1.05 : 1. The mean IC50 values in an activity assay for rAAT and rAAT-Alexa488 were 4.9 nM and 6.5 nM, respectively, showing no significant change in activity after the rAAT was conjugated with Alexa488.

### 3.3. Tissue Localization of rAAT-Alexa488 and Acetyl-*α*-Tubulin

**Figure 3:**
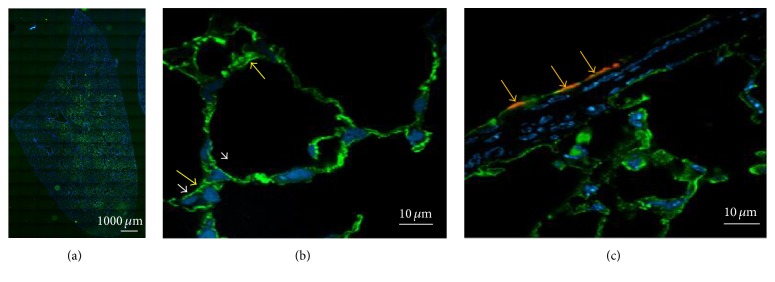
Localization of rAAT-Alexa488 in the lung following single IT administration of 5 mg/kg rAAT-Alexa488 to ALI-induced mice. Immunofluorescence microscopy, showing rAAT-Alexa488 (green), acetyl-*α*-tubulin (red; (c) only), and cell nuclei labeled with DAPI (blue). rAAT-Alexa488 is primarily centered around large airways (a). Higher magnification shows that rAAT-Alexa488 is primarily distributed to the surface of pneumocytes (white arrowheads) lining alveoli and to interstitial spaces (yellow arrows) within lung parenchyma (b). The presence of rAAT-Alexa488 on the mucosal surface of large airways is confirmed by dual immunofluorescence with acetyl-*α*-tubulin, which is expressed by ciliated respiratory epithelial cells (orange fluorescence, yellow arrows) (c).

Fluorescence microscopy was used to localize rAAT-Alexa488 in mouse lung following IT administration and revealed that rAAT-Alexa488 biodistribution was centered around large airways (Figure 3(a)). Higher magnification showed that rAAT-Alexa488 was localized primarily to the surface of epithelial cells lining large airways and to the surface of alveolar pneumocytes as well as to interstitial spaces within the alveolar septa (Figure 3(b)). The presence of rAAT-Alexa488 on the mucosal surface of large airways in the lung was confirmed by dual immunofluorescence for acetyl-*α*-tubulin, which labels microtubules in the cilia located on the apical aspect of respiratory epithelial cells that line large airways (Figure 3(c)).

### 3.4. Neutrophil Localization in Lungs of LPS/fMLP-Induced ALI Mouse Model

**Figure 4:**
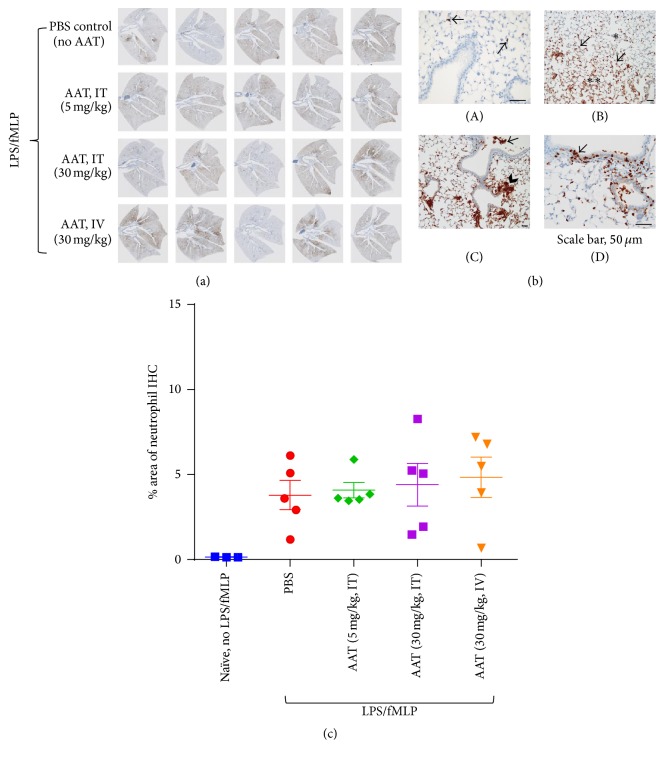
IHC using antibody 7/4 to localize neutrophils in lung sections. The numbers and patterns of distribution of Ab7/4-positive neutrophils are demonstrated through localization of the brown chromogen diaminobenzidine (DAB). (a) Lung sections (*N* = 5, 5 animals per group) following single IT or IV administration of PBS, 5 or 30 mg/kg of rAAT to ALI-induced mice. (b) (A) Section of lung from the naïve mouse group (no LPS/fMLP). Arrows identify individual Ab7/4-positive cells (brown DAB chromogen) within alveolar walls. (B) Section of lung from rAAT 30 mg/kg IV group after LPS/fMLP insult, showing interface (arrows) between regions of moderate (*∗*) and marked (*∗∗*) numbers of Ab7/4-positive neutrophils. (C) Section of lung from PBS group LPS/fMLP, showing Ab7/4-positive neutrophils in airways (arrow) and dense foci of Ab7/4-positive neutrophils adjacent to airways (arrowhead). (D) Section of lung from rAAT 30 mg/kg IT group after LPS/fMLP administration, showing Ab7/4-positive neutrophils transmigrating across respiratory epithelium lining large airway. (c) Quantitative IHC (QIHC) for Ab7/4-positive neutrophils in lung sections following single IT or IV administration of 5 or 30 mg/kg of rAAT to ALI-induced mice.

Neutrophil localization by IHC using a specific antibody (Ab7/4) in sections of lung from naïve mice and mice following single IT or IV administration of PBS or rAAT in LPS/fMLP-induced ALI mice (NE680 probe study) are shown in Figures 4(a) and 4(b). Numbers of neutrophils present in the lung parenchyma among groups were quantified by QIHC (Figure 4(c)). Small numbers of evenly dispersed Ab7/4-reactive neutrophils were diffusely scattered throughout the pulmonary parenchyma of naïve mice (Figure 4(b)(A)). Among naïve mice, immunoreactive cells occurred exclusively as individual cells within alveolar septa and were not found within airways or alveolar lumens and were rarely localized in intraepithelial sites within the respiratory mucosa lining airways. In contrast, despite variability among animals within dose groups, significantly greater numbers of Ab7/4-positive neutrophils were observed in the pulmonary parenchyma of all mice that received LPS/fMLP as compared with naïve mice (Figures 4(b)(B), 4(b)(C), and 4(b)(D)). There was a general increase in the density of Ab7/4-positive neutrophils throughout the lung parenchyma of LPS/fMLP treated mice, with multifocal to coalescing sites containing markedly increased numbers of immunoreactive cells (Figure 4(b)(B)). Ab7/4-immunoreactive cells were frequently localized within the lumens of alveoli and airways and often occurred in small, dense foci adjacent to airways (Figure 4(b)(C)) and could be seen transmigrating through respiratory epithelium lining airways (Figure 4(b)(D)). Overall, there was no difference in the pattern of distribution of Ab7/4-positive cells among dose groups that received LPS/fMLP, nor was there a significant difference in the numbers of neutrophils present in the lung parenchyma among groups as quantified by QIHC (Figure 4(c)).

### 3.5. Tissue Localization and Image Analysis of Activated NE680 by Neutrophil Elastase in the Presence or Absence of rAAT in LPS/fMLP Mice

**Figure 5:**
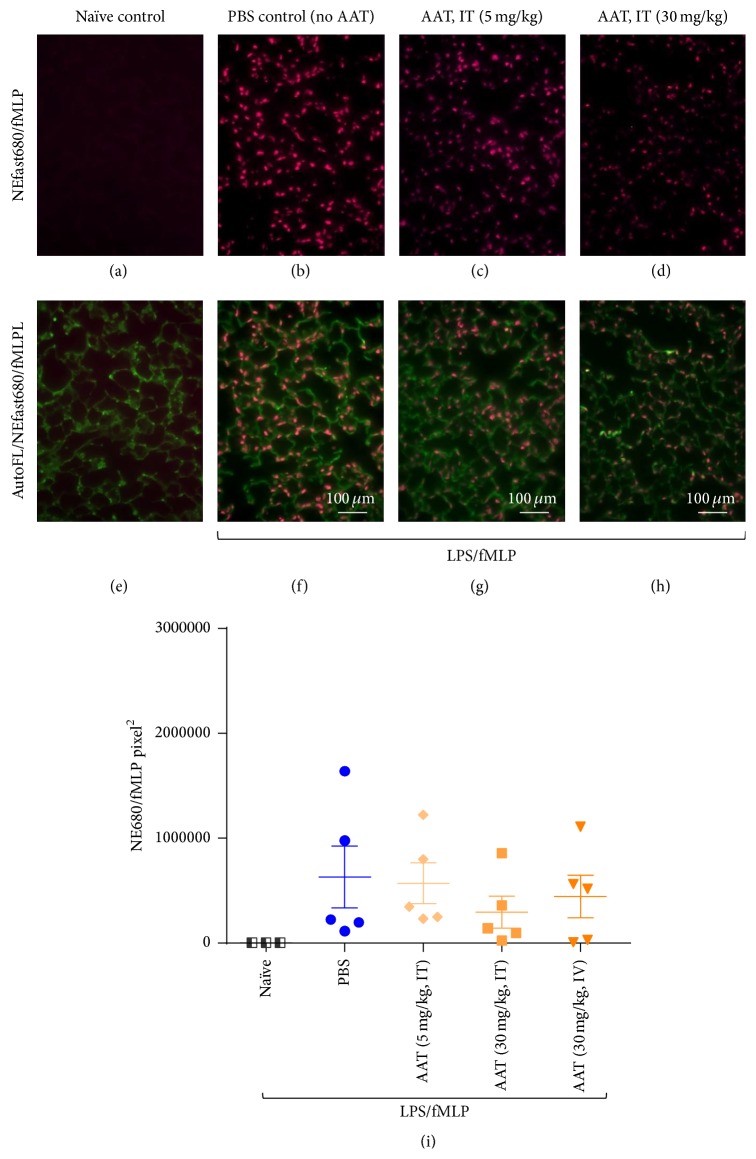
Localization of neutrophil elastase activity by NE680 fluorescence in lung sections from naïve mice (a and e) or following single IT administration of PBS (b and f) or 5 mg/kg (c and g) or 30 mg/kg (d and h) of rAAT to ALI-induced mice. Panels show NE680 signal expression (red fluorescence, (a)–(d)) and in the context of lung tissue morphology, as revealed by autofluorescence (green fluorescence, (f)–(h)). (i) Measurement of neutrophil elastase activity by image analysis quantification of NE680 fluorescence in lung sections following single IT or IV administration of 5 or 30 mg/kg of rAAT to ALI-induced mice. Results are shown as mean ± SEM (*N* = 5 animals per dose group). There were no significant differences among groups based on one-way ANOVA (Kruskal-Wallis test with Dunn's multiple comparisons test; *p* = 0.608).

As expected, no NE680 fluorescence signal was detected in the lungs of naïve mice (Figures 5(a) and 5(e)), while fluorescence was observed in the lungs of all LPS/fMLP-induced mice after IT injection of NE680, indicating activation of NE680 probe by neutrophil elastase following induction of ALI (Figures 5(b)–5(d) and 5(f)–5(h)). Activated NE680 probe was localized primarily to cells within septa and lumens of alveoli and to a lesser extent to the extracellular space within the alveolar interstitium (Figure 5). The group mean value for NE680 fluorescence, measured by QIHC, was lowest in the group that received 30 mg/kg of rAAT by IT administration (Figure 5(i)); however, the differences among groups were not statistically significant by one-way ANOVA (Kruskal-Wallis test with Dunn's multiple comparisons test; *p* = 0.608).

## 4. Discussion

The administration of rAAT by inhalation has gained interest as an alternative to intravenous augmentation therapy to treat the lung disease associated with AAT deficiency. A principal goal of using inhalation as the route of administration is to improve the biodistribution of AAT to the lungs of AAT-deficient patients, thereby reducing the dose required to restore the protease/antiprotease (NE/AAT) balance and diminishing the detrimental effects of excessive NE activity in the lungs. Since the precise site of action of AAT and the concentration required for efficacy at the site of action after inhalation are not well understood, it is hard to accurately predict efficacious human doses for administration by this route. Preliminary predictive modeling studies indicated that the site of action is in the pulmonary interstitium and epithelium lining fluid and that sufficient concentrations of functional AAT can inhibit NE and lead to rescue of AAT deficiency [18]. This investigation was aimed at evaluating rAAT distribution and localization after inhalation. Intratracheal (IT) administration, the direct instillation of a test article into the lungs via the trachea, has been employed in many studies as an alternative exposure procedure to inhalation [19]. IT administration is simpler than inhalation exposure in rodents and permits the introduction of a range of doses to the lungs within a short time, although the distribution of instilled material within the respiratory tract will likely differ from the distribution of inhaled material. Our investigation chose to use IT as the route of administration to assess drug deposition in mouse lungs.

To estimate the concentration of rAAT at the site of action, a series of in vivo studies were conducted. A PK and tissue distribution study using [^125^I]rAAT, carried out to study distribution in lung in relationship to serum, demonstrated that rAAT exposures in the lungs were significantly higher following IT administration compared to those observed following IV dosing, but the distributions in other organs were similar between the two routes of administration.

Analysis of lung sections by fluorescence microscopy following IT administration of rAAT-Alexa488 in an ALI-induced mouse model revealed local deposition of rAAT in lung. rAAT-Alexa488 was mainly deposited to the surface of epithelial cells lining large airways, to the surface of pneumocytes lining alveolar lumens, and to interstitial spaces within the alveolar parenchyma (areas which are predicted to be the site of action) but not to intracellular locations within alveolar pneumocytes.

NE680, a specific NE molecular imaging agent developed to detect and quantify NE activity in vivo [13], was utilized in this investigation to examine rAAT function at the site of action in lungs in an ALI-induced mouse model. Since AAT is an inhibitor of NE, administration of rAAT was expected to reduce NE680 signal through inactivation of NE activity. To this end, we first reproduced the ALI model in our lab by challenging mice by intratracheal administration of LPS and fMLP, which act synergistically to cause lung inflammation via massive neutrophil infiltration and degranulation [14, 15]. Ab7/4-immunoreactive cells (neutrophils) were frequently localized within the lumens of alveoli and airways, often occurred in small, dense foci adjacent to airways, and could be seen transmigrating through respiratory epithelium lining airways. The heterogeneous distribution pattern of Ab7/4-positive neutrophils is very likely related to the IT delivery method, and it was reported that instillation would result in heavier and more centralized particle deposition, likely due to the bolus delivery, whereas inhalation resulted in a wider and more even distribution or particles throughout the lung [19]. We did not observe a reduction in numbers of neutrophils in the lungs of mice dosed with rAAT in this study, which stands in contrast to the findings of Jonigk and colleagues [20]. The differences in effect of rAAT administration on LPS-induced pulmonary neutrophil infiltration in these two studies are likely due to distinct differences in the experimental designs of these experiments. Jonigk et al.'s paper describes a prophylactic study, in which mice were dosed with AAT or rAAT before LPS was administered. In contrast, our study used a therapeutic approach, in which mice were dosed with LPS >17 hours before rAAT was administered. Thus, neutrophil recruitment and infiltration into the lung parenchyma were well established before rAAT therapy was initiated in our study. As pointed out in Jonigk et al.'s manuscript, AAT has been shown to suppress inflammation and immunomodulatory pathways in addition to inhibiting elastase activity; moreover, their study provides proof of the anti-inflammatory effects of AAT by demonstrating the anti-inflammatory effects of a recombinant rAAT that lacks antielastase activity. Thus, one would expect greater efficacy of rAAT in preventing neutrophil recruitment and infiltration in Jonigk et al.'s study when compared to ours. Other differences between these two studies included our use of fMLP to induce degranulation of neutrophils and completely different methods of neutrophil quantification; in their study, neutrophils are quantified in BAL fluid, whereas our study quantified neutrophils in lung tissue sections by quantitative immunohistochemistry. Through NE680 probe detection, NE activity, following LPS/fMLP induction, was found to localize within cells in alveoli and alveolar septa and less within the alveolar interstitium (Figure 5). The distribution of NE was not uniform within the lung parenchyma and the intersubject variability was high. As for Ab7/4-positive neutrophils, an uneven distribution of the NE680 signal was observed throughout the lung following IT administration, despite careful and slow instillation. NE680 did not correlate with the pattern of neutrophil localization as determined by IHC possibly due to variability in distribution of LPS, fMLP, and NE680 probe in the lung following separate IT administrations, which lead to multifocal induction and distribution of neutrophils and the NE680 probe.

Recombinant AAT was administered at 5 and 30 mg/kg IT in this ALI mouse model to assess its inhibitory effect in vivo. Image analysis quantification of lung sections revealed that the mean value for NE680 fluorescence was lowest in the group that received 30 mg/kg rAAT by the IT route (Figure 5). There was no difference in the pattern of distribution of Ab7/4-positive cells among treatment groups following LPS/fMLP insult (PBS or rAAT; Figure 4), nor was there a significant difference in the numbers of neutrophils present in the lung parenchyma among treatment groups as quantified by QIHC (Figure 4).

It has been reported that the amount of material deposited within the alveolar region relative to the bronchial region of the rat lung is greater with inhalation than with IT instillation [21] and that inhalation resulted in greater deposition in apical areas of the lungs compared with basal areas, while the deposition pattern was opposite following IT instillation [22]. Despite the difference between inhalation and IT administration, our study using the ALI mouse model demonstrated that NE is mainly localized within the alveolar interstitium and within cells throughout the pulmonary parenchyma. Following a single IT administration of rAAT, NE in lung tissues of LPS/fMLP-induced ALI mice was suppressed in a dose-dependent fashion from 5 mg/kg to 30 mg/kg compared to the PBS treated group. The conservative concentration estimate of rAAT at the site of action will improve confidence in projecting the inhalation dose for first in human studies.

## Abbreviations

AAT: Alpha-1 antitrypsin
ALI: Acute lung injury
IT: Intratracheal
LPS/fMLP: Lipopolysaccharide/fmet-leu-phe
NE680: Neutrophil elastase 680 FAST.
